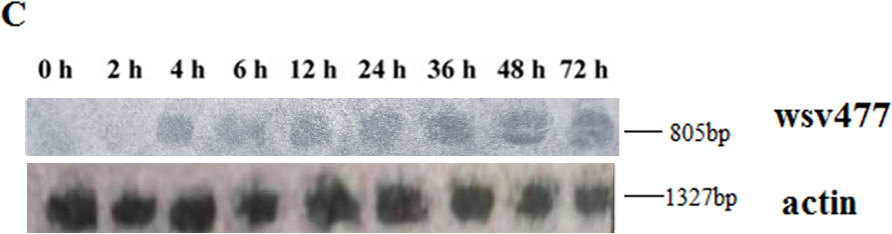# Correction for He et al., “Viral MicroRNAs Targeting Virus Genes Promote Virus Infection in Shrimp *In Vivo*”

**DOI:** 10.1128/jvi.02106-24

**Published:** 2025-01-24

**Authors:** Yaodong He, Kai Yang, Xiaobo Zhang

## AUTHOR CORRECTION

Volume 88, no. 2, p. 1104–1112, 2014, https://doi.org/10.1128/jvi.02455-13. Page 1105: Figure 1C should appear as shown in this correction. In the original article, an incorrect image was inadvertently used for the wsv477 control. We deeply regret this error and sincerely apologize for any inconvenience to the readers.

**Fig 1:**